# Alexithymia and Autistic Traits as Contributing Factors to Empathy Difficulties in Preadolescent Children

**DOI:** 10.1007/s10803-021-04986-x

**Published:** 2021-03-31

**Authors:** Lydia Gabriela Speyer, Ruth Harriet Brown, Lorna Camus, Aja Louise Murray, Bonnie Auyeung

**Affiliations:** 1 Psychology Department, The University of Edinburgh, 7 George Square, Edinburgh, EH8 9JZ UK; 2 Psychology Department, Heriot Watt University, Boundary Road North, Edinburgh, EH14 4AS UK; 3 Autism Research Centre, Department of Psychiatry, University of Cambridge, Cambridge, CB2 8AH UK

**Keywords:** Empathy, Alexithymia, Autistic Traits, Children, Multi-Informant Approach

## Abstract

Recent evidence suggests that, contrary to traditional views, empathy difficulties may not be a core feature of autism; but are rather due to co-occurring alexithymia. Empathy, alexithymia and autistic traits have yet to be examined concurrently in children. Therefore, we examined the co-occurrence of empathy difficulties and alexithymia in 59 typically developing and 5 autistic children. Multiple measures (self-report, parent-report and a behavioural task) were used to evaluate empathy and to assess differences in self- and parent-reports using multiple regressions. Alexithymia was found to predict empathy significantly better than autistic traits, providing support for the alexithymia hypothesis. From a therapeutic perspective, results suggest autistic children who screen positive for elevated alexithymic traits may benefit from additional support targeting emotion identification.

It has long been assumed that autism is associated with difficulties in empathy (Charman et al., 1997; Hobson, 1986). Indeed, numerous past questionnaire-based (e.g. Johnson et al., 2009; Lombardo et al., 2007) and neuroimaging (e.g. Greimel et al., 2010; Schulte-Ruther, 2011) studies have identified significant differences in empathic abilities between autistic individuals and neurotypical counterparts. However, there have been growing calls for research in autism to both view empathy as a multi-faceted, not unidimensional, psychological construct (Mul et al., 2018) and to consider potential underlying factors in these empathy difficulties such as alexithymia (Bird & Cook, 2013).

Fletcher-Watson and Bird (2019), for example, recently proposed a four-stage empathy model, which progresses from noticing the outward emotional cue of another (stage one) to choosing the socially appropriate empathic response (stage four). The authors speculated that autism is not associated with a global empathy ‘impairment’; rather, autistic adults and children may experience strengths and challenges along this multifaceted empathy model. For example, an autistic individual may feel a strong sense of empathy towards an upset person, but may find it difficult to choose the correct response that may be heavily influenced by complex societal norms (Fletcher-Watson & Bird, 2019). Supporting this, autistic individuals have reported that while they do indeed feel empathy for others, some can feel they ‘under-’ or ‘over-express’ their emotional reactions which in turn may be misconstrued as unempathetic by non-autistic individuals (Russell et al., 2019). Thus, it is unsurprising that there has been significant heterogeneity within the past research investigating the link between autism and empathy (Uljarevic & Hamilton, 2012). For example, similar behavioural (e.g. emotion recognition; Bird & Cook, 2013) and neurological (e.g. brain activity when viewing others in pain; Hadjikhani et al., 2014) empathic responses have been observed between autistic and neurotypical individuals.

One possible explanation for the conflicting findings in research investigating the relationship between autism and empathy is the ‘alexithymia hypothesis’ (Bird & Cook, 2013). Alexithymia, defined as marked difficulties in identifying and describing one’s own emotions, is a subclinical phenomenon that often co-occurs with autism (Sifneos, 1973), and is related to decreased empathic behaviours, even within neurotypical samples (Grynberg et al., 2010). Alexithymia occurs in approximately 10% of the general population (Mattila et al., 2006) and as many as 50% of the autistic population (Hill et al., 2004; Lombardo et al., 2007). It has been proposed that many of the psychosocial difficulties associated with autism, such as empathic responses (Bird et al., 2010), emotion recognition (Cook et al., 2013) and eye-fixation (Bird et al., 2011) difficulties are not a characteristic of autism itself, but rather a characteristic of co-occurring alexithymia (Bird et al., 2010; Fletcher-Watson & Bird, 2019; see Kinnaird et al., 2019 for review). Furthermore, an association between decreased empathy task performance and difficulty perceiving one’s internal bodily, visceral and emotional states (i.e., interoceptive awareness; Herbert et al., 2011) has been found to be explained by co-occurring alexithymic traits in individuals with ASC (Silani et al., 2008). Building upon the original alexithymia hypothesis (Bird & Cook, 2013) and the work in interoceptive awareness, Bird and Viding (2014) proposed the Self to Other Model of Empathy (SOME) to provide a framework for understanding the differing contributions of autism and alexithymia on empathy difficulties. The SOME posits that multiple cognitive processes are responsible for empathic abilities; notably the ‘situation understanding system’ (i.e., the processes that lead to an individual determining the emotional state of another based on the situation they are in) and the ‘affective representation system’ (i.e., the processes which lead the individual to form representations of their own, and others’, emotional states). The authors concluded that alexithymia is associated with global emotion representation and recognition difficulties as a result of impairments within the affective representation system, potentially as a consequence of interoceptive awareness deficits. Empathy difficulties in autism however were concluded to be a consequence of impairment in the situation understanding system, potentially underpinned by theory of mind (ToM) deficits, but only when these ToM deficits are severe (Bird & Viding, 2014).

Recent results investigating the alexithymia hypothesis have, however, not been consistent. For example, a newly-developed experimental paradigm developed by Santiesteban and colleagues (the Continuous Affective Rating and Empathic Responses (CARER) Task; in press) was constructed to measure various facets of empathy, including emotion recognition, affective empathy (i.e., the degree to which one’s emotional state matches to the emotional state of another), and affective sharing (i.e., correctly matching one’s emotional state to the state that was attributed to another). After controlling for alexithymia, no deficits in affect sharing were observed in the autistic individuals, however, some difficulties remained in retrospectively inferring the emotional state of others, potentially influenced by ToM deficits (Bird & Viding, 2014). Thus, supporting the speculations of Bird and Cook (2013), Bird and Viding (2014), and Fletcher-Watson and Bird (2019), the authors concluded that autism is not associated with diminished empathic abilities. Rather, alexithymia appeared to underpin difficulties in some of the important cognitive processes required for empathy, particularly sharing the affective state of others (Santiesteban et al., 2020). Contrastingly, in a questionnaire-based study by Shah and colleagues (2019) in neurotypical adults, regression analyses found that the significant relationship between alexithymia and self-reported empathy difficulties diminished after adjusting for autistic traits. Considering the heterogeneity of the past literature, additional research on the alexithymia hypothesis is required prior to extrapolating the findings to clinical practice.

Findings on empathic abilities in ASC child populations have also been heterogeneous. For example, while children with ASC have been found to react less empathically towards emotional vignettes compared to matched controls (Yirmiya et al., 1992), similar empathic reactions in response to other emotional stimuli have been observed in ASC and non-ASC children (Capps et al., 1993). Thus, similar to the speculation in adult populations, these contradictory results may be a consequence of the children’s co-occurring alexithymia. Indeed, children with ASC have been found to have significantly elevated levels of alexithymia compared to children without ASC (Griffin et al., 2015; Trevisan et al., 2016). Given these findings, additional work is required in child populations to examine the role of potentially co-occurring alexithymia in the heterogeneous emotional difficulties that are typically attributed to autism. To date, no study has been conducted to investigate this.

A consistent limitation of the past investigations has been the reliance on self-assessed psychometric measures of alexithymia and empathy (e.g. Shah et al., 2019). This poses an inherent problem, where heightened alexithymic traits may inhibit the correct identification and reporting of one’s emotional understanding, or lack thereof. Further, it has been suggested this decrease in metacognition due to alexithymic traits may be further exacerbated in younger populations (Myers & Winters, 2002). While attempts have been made to circumvent this by using self- and parent-reports of alexithymia concurrently, they have previously been found to correlate non-significantly (r = − 0.040; Griffin et al., 2015). Considering this, it is possible that children and their parents have varying degrees of insight into the child’s emotional difficulties and thus utilise different information when rating alexithymic traits (Brown, Murray, Stewart and Auyeung, 2020). Likewise, empathic behaviour has typically been measured solely with parent-reports in children (e.g. Mensi et al., 2018). This single-measurement method may be insufficient to assess discrete types of empathy (e.g. empathic traits and empathic behaviours towards others; Hall & Schwartz, 2019). Furthermore, parents may only be able to rate their child’s observable empathic abilities (e.g. empathic behaviours) and may thus have a limited perspective on their child’s co-occurring empathic thoughts, beliefs and motives (Murphy, 2019).

Thus, there have been growing calls for researchers to use multi-method assessments when investigating empathic behaviour, particularly by incorporating experimental empathy tasks with psychometric assessments (Murphy & Lilienfeld, 2019; Santiesteban et al., in press). The Kids’ Empathic Development Scale (KEDS; Reid et al., 2013) is one such task, which assesses empathy across a series of processes; the child’s ability to explain and justify the emotional reaction of an individual (i.e., cognitive empathy), to take the place of the individual and envisage what emotion(s) the person is feeling (i.e., affective empathy), and to provide a hypothetical pro-social reaction to the given situation (i.e., behavioural empathy). Therefore, addressing the limitations of the past research, the current study will be the first to investigate the relationships between empathy, autistic traits and alexithymia in children, as assessed using both the KEDS and a battery of self- and parent-reported measures.

## The Current Study

The current study will investigate the co-occurrence of empathy difficulties and alexithymia in typically developing children and a small sample of autistic children, using the Autism Spectrum Quotient for Children (AQ-Child) (Auyeung et al., 2008). The study will also assess differences in self-reported empathy in children with alexithymia compared to parent-reports and the KEDS behavioural task. We hypothesised that any significant association between AQ-Child and empathy abilities will attenuate to non-significant when adjusting for alexithymia. Furthermore, higher alexithymia scores are expected to be associated with less advanced empathy abilities.

## Methods

### Sample Size

A sample size of 60 children was chosen as a priori power analysis suggested that for 5 predictors, power level of 0.8 and a significance level of p < 0.05, 39 participants was required. This was conducted based on the effect sizes of previous studies analysing alexithymia (e.g. Bird et al., 2010). To allow for missing data and for better detectability of effects, a final sample of 60 was deemed adequate.

### Participants

The participants of this study were 59 typically and atypically developing children aged 8 to 12 (33 male, M_age_ = 9.46, SD = 1.28). The parents of five children (4 boys, 1 girl) reported their child had an Autism Spectrum Condition (ASC). As increasing evidence suggests autistic traits fall on a spectrum (Abu-Akel et al., 2019; Robinson et al., 2011), the autistic children were considered to fall on the extreme end of this continuum. Confirming this, the neurotypical children within the sample were found to have an average AQ score of 52.89 (SD = 5.93), whereas the autistic children had an average AQ score of 95.20 (SD = 23.84). Thus, to capture a wide range of autistic traits, alexithymia and empathy difficulties, the autistic children were included in the study (see Table 1 for a full overview of the score differences between the autistic and non-autistic children). A total of 33 children were male, and 26 children were female. All children were fluent in English. The participants were recruited through the University of Edinburgh’s Developmental Lab’s subject pool. Before participating in the study, parent(s) gave informed consent and children were asked to assent in accordance with the British Psychological Society Code of Human Research Ethics (BPS, 2014). This study was approved by the University of Edinburgh’s ethics committee (210-1718/1).

**Table 1:** Means and Standard Deviations for Continuous Predictors

Variable	Autistic Children			Neurotypical Children			All Children		
	Mean	SD	Range	Mean	SD	Range	Mean	SD	Range
Age	10.20	0.84	9–11	9.39	1.30	8—12	9.46	1.28	8 – 12
KEDS	62.80	18.18	31–77	73.85	14.22	21—104	72.90	14.74	21 – 104
EmQue-CA	24.60	2.30	22–27	26.56	4.44	14—34	26.39	4.33	14 – 34
EQ	21.40	6.03	16–31	35.13	10.37	8—51	33.95	10.76	8 – 51
AQ-Child	95.20	5.93	89–102	52.89	23.84	7—124	56.54	25.78	7 – 124
IQ	100.20	7.95	92–112	108.40	12.68	83—140	107.71	12.51	83 – 140
AlexQ-CP	43.80	3.35	40–48	33.87	6.50	23—47	34.73	6.87	23 – 48
AlexQ-C	39.36	5.63	35–49	37.85	4.63	28—48	37.98	4.69	28 – 49

KEDS: Kids Empathic Development Scale; EMQue-CA: Empathy Questionnaire for Children and Adolescents; EQ: Empathy Quotient; AQ – Child: Autism Quotient – Child; IQ: Intelligence Quotient; AlexQ-CP: Alexithymia Questionnaire for Children – Parent; AlexQ-C: Alexithymia Questionnaire for Children

### Measures

The following measures were used in the present study and were administered as part of a larger battery of questionnaires and behavioural tasks:

#### Wechsler Abbreviated Scale of Intelligence–II (WASI)

In order to adjust for any differences in empathy abilities that could be due to an above or below average IQ, the vocabulary and matrices subsets of the WASI-II were used to calculate full-scale IQ. The WASI has frequently been used in children with and without ASC and has shown high reliability (Minshew et al., 2005).

#### Autism Spectrum Quotient-Child – Parent Questionnaire (AQ-Child)

The child version of the AQ (Baron-Cohen et al., 2001) is a 50-item parent-report questionnaire that was developed to measure autistic traits in children aged 4 to 11 years old (e.g. “S/he prefers to do things the same way over and over again”). The AQ-Child items are measured on a 4-point Likert scale (‘definitely agree’, ‘slightly agree’, ‘slightly disagree’ and ‘definitely disagree’) with a score range of 0–150. Higher scores are indicative of more pronounced autistic traits. The AQ-Child has shown to have high sensitivity (95%), specificity (95%) and good test–retest reliability (Auyeung et al., 2008). Internal consistency of the AQ-Child scores were found to be excellent in the current study (α = 0.950).

#### Alexithymia Questionnaire for Children (AlexQ-C)

The AlexQ-C is a 20-item self-report questionnaire measuring the core facets of alexithymia: difficulties in identifying feelings, difficulties in describing feelings and externally oriented thinking (e.g. “I am often confused about the way I am feeling inside”). It is rated on a 3-point Likert Scale (‘not true’, ‘sometimes true’, ‘often true’) with scores ranging from 0 to 40. Higher scores indicated higher levels of alexithymic traits. The AlexQ-C scores have been previously shown to have good internal consistency (α > 0.750) in a sample of 9 to 15-year-old children (Rieffe et al., 2006). The AlexQ-C scores in the current study were found to be satisfactory (α > 0.610).

#### Alexithymia Questionnaire for Children – Parent Version (AlexQ-CP)

The AlexQ-CP is an adaptation of the AlexQ-C and has been successfully used to assess alexithymia in children aged 3 to 13 (Costa et al., 2017; e.g. “My child is often confused about the way they feel inside”). Like the AlexQ-C, higher scores are indicative of more pronounced alexithymic traits, with a score range of 0 to 40. Internal consistency of the AlexQ-CP in the current study was found to be good (α > 0.850), similar to the findings of Costa and colleagues (α > 0.860; Costa et al., 2017).

#### Empathy Questionnaire for Children and Adolescents (EmQue-CA)

The EmQue-CA is an 18-item self-report empathy questionnaire (e.g. “When a friend is upset, I feel upset too”). Children rate statements on their empathy on a 3-point Likert scale (‘not true’,’sometimes true’ and’often true’) with scores ranging from 0 to 36. Higher Scores indicate more advanced empathic abilities. The EmQue-CA has shown high convergent validity and high internal consistency (α > 0.700; Overgaauw et al., 2017). Here, the EmQue-CA scores were found to be slightly higher (α > 0.750).

#### Empathy Quotient Child – Parent Questionnaire (EQ-Child)

The Child version of the EQ is a 27-item parent-report questionnaire that was developed for children aged 4 to 11 (e.g. “My child likes to look after other people”). Parents are asked to rate statements on their child’s empathy on a 4-point Likert scale (‘definitely agree’, ‘slightly agree’, ‘slightly disagree’, or ‘definitely disagree’), with scores ranging from 0–54. Higher scores are indicative of more empathic behaviour. The measure has shown good test–retest reliability and high internal consistency (α = 0.900; Auyeung et al., 2009), with the current study finding similar results (α = 0.900).

#### Kids Empathic Development Scale (KEDS)

The Kids Empathic Development Scale is a behavioural empathy task that is used to measure complex emotions and mental state comprehension in children aged between 7 and 10 (Reid et al., 2013). The KEDS consists of one sample picture and twelve test pictures that show children in various individual and interpersonal situations of different social complexity. On each of the pictures, one or two children’s faces are blanked out. First, to assess affective empathy, the child is requested to use affective inference to ascribe one of six emotions (i.e., happy, sad, angry, relaxed, surprised, afraid) to the children with the blank faces (e.g. “If you were that child, how would you feel?”). To account for more complex emotions, participating children are informed they can answer with more than one target emotion. As a follow-up question, to measure cognitive empathy, the child is then asked why they think the child is feeling this specific emotion and what they would do if they were in that specific situation (e.g. “Can you tell me why this child is feeling that way?”). Finally, as a control question to check whether the child understood the picture, they are asked to offer some details on the situation that is depicted.

### Procedure

Prior to participation, children and their parent(s) were informed of the nature of the study and provided with an information sheet. Parents were then asked to sign a consent sheet and children asked to give assent to taking part in the study. After giving assent, children were invited to come into a quiet room to complete the battery of behavioural tasks, the WASI and the questionnaire booklet. The parent(s) of the children were asked to remain in the waiting area and complete the parent-appropriate questionnaire pack. One researcher stayed with the child during the procedure while a second researcher assisted with task administration. On completion, parent(s) and their children were compensated monetarily for their time and debriefed on the study’s main objectives. Participation lasted a maximum of one and a half hours.

### Design and Statistical Analysis

This study used a cross-sectional between-subject design for analysing the relationship between empathy, alexithymia and autistic traits. In order to collapse both the empathy and alexithymia measures into singular outcome variables for the first regression model, indices were calculated. This was done by averaging the scaled measures of the respective constructs (i.e., EQ, EMQue-CA, and KEDS scores for the empathy index; and AlexQ-C and AlexQ-CP scores for the alexithymia index). For better interpretability, all continuous predictors were transformed into z-scores. Hierarchical linear regression models with the empathy index as the outcome, the covariates IQ, age and gender entered in the first step and alexithymia index and autistic traits entered in the second step was fitted to analyse this relationship. Models were trimmed to only include significant predictors using F-tests for model comparisons. Next, the three different empathy measures were analysed separately in further hierarchical regression models to be able to better understand the different forms of measuring empathy. Similarly, the two alexithymia measures were also used separately in the latter regression models to better understand the contributions of self- and parent-reported alexithymic traits. The statistical analysis was performed using the open-source software R (R Core Team, 2017) and R Studio (RStudio Team, 2016).

## Results

### Descriptive Statistics

**Fig. 1:**
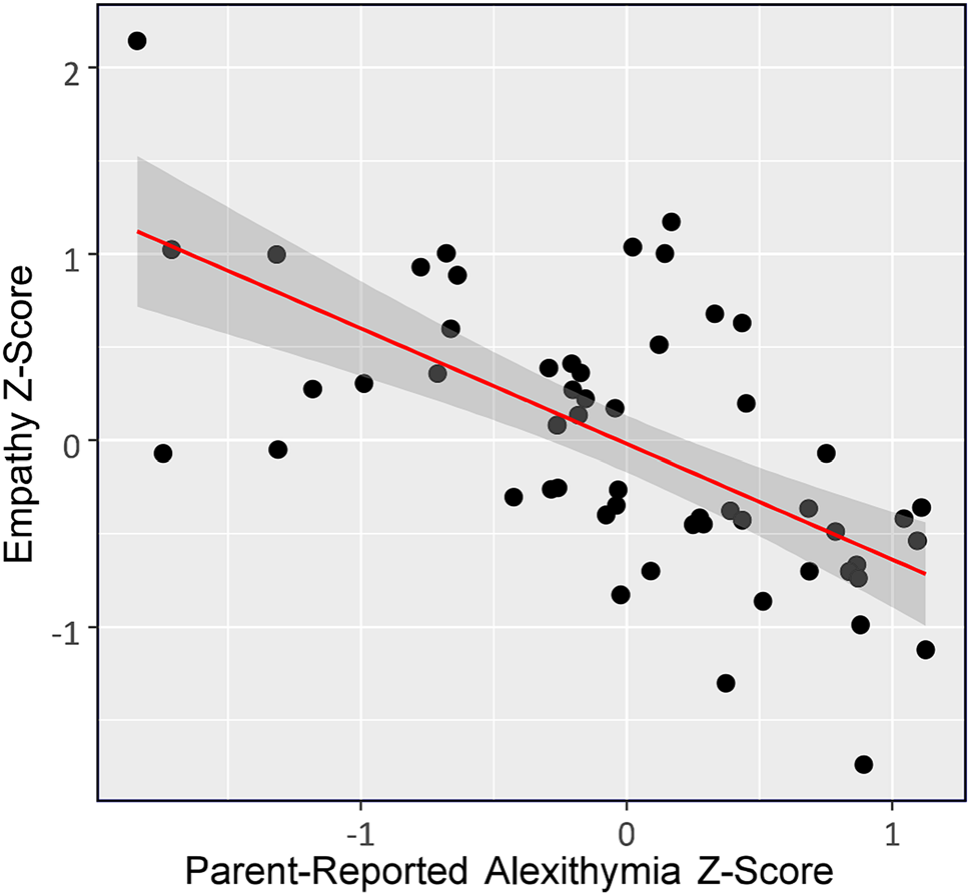
Scatterplot visualising the relationship between empathy and alexithymia

**Fig. 2:**
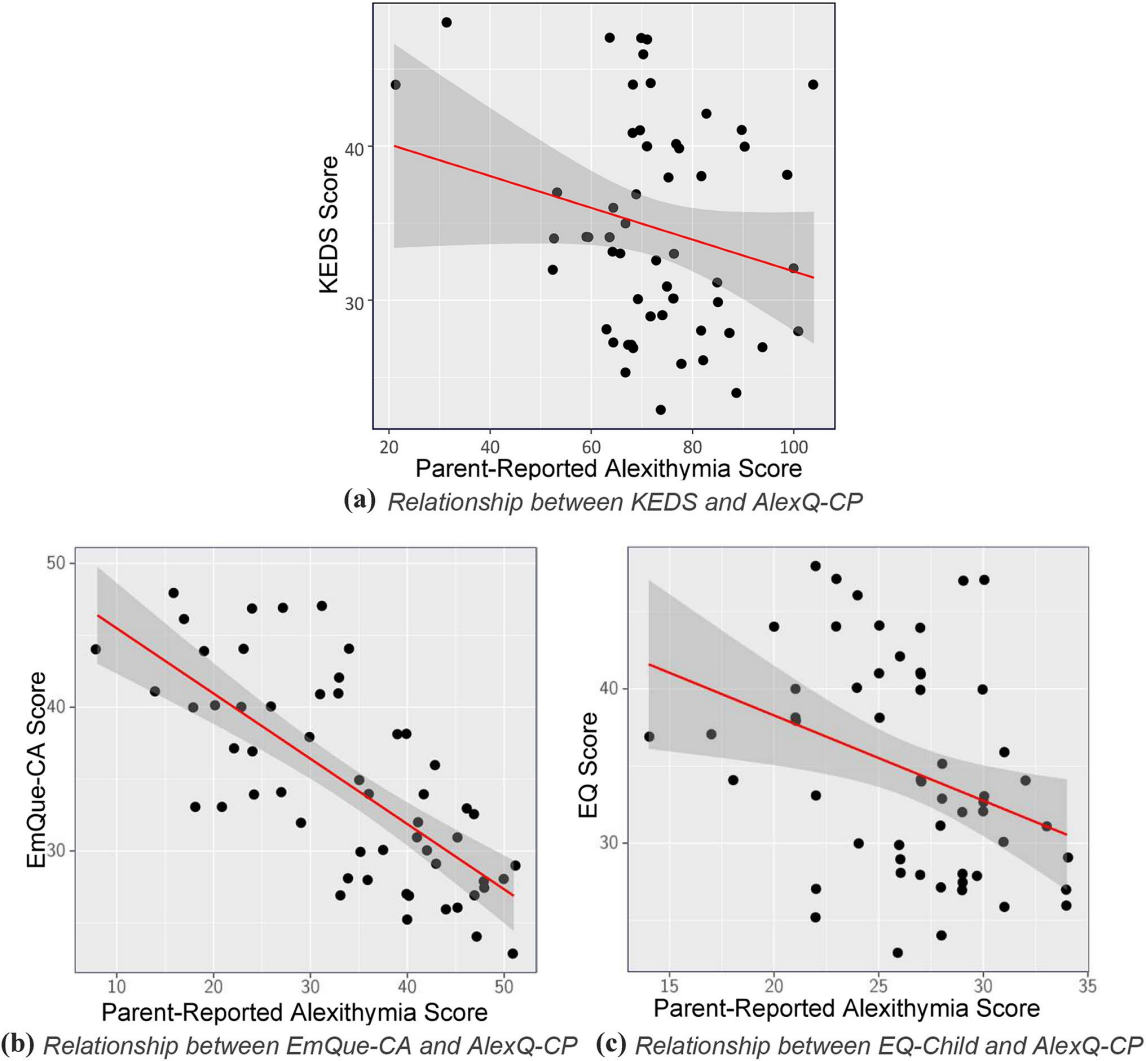
Scatterplots visualising the negative relationship between the three different measures of empathy (Empathy Questionnaire for Children and Adolescents (EmQue-CA), Empathy Quotient Child–Parent Questionnaire (EQ-Child), Kids Empathic Development Scale (KEDS)) and parent-reported alexithymia as measured by the Alexithymia Questionnaire for Children – Parent Version (AlexQ-CP)

Prior to the main analysis, the data were cleaned and the variables were operationalised. An a priori missing data rule was implemented, in which if < 4 items in a measure were missing, these items were replaced with the mean value of the other items of the questionnaire. If > 4 items were missing, the data were not used for analysis, which led to the exclusion of 4 participants from some but not all of the measures. In addition, one participant was completely removed as they did not fulfil the age requirement of 8 to 12. This resulted, depending on the measures used for the respective models, in a final sample size of 55 to 59 children. Descriptive statistics are given in Table 1. The relationship between alexithymia and overall empathy is visualised in Fig. 1 and a breakdown of this relationship for each empathy measure is given in Fig. 2.

Data were additionally checked for univariate and multivariate outliers using Mahalanobis distance, for multivariate normality using Mardia’s multivariate normality test, and for multicollinearity to ensure that a multiple linear regression was indeed the appropriate analysis for the data. A correlation matrix, with the full sample, is presented in Table 2. To confirm that the significant correlations were not due to including the autistic children in the sample, the correlation matrix was rerun using solely typically developing children (see Table 3). After conducting multiple regressions, diagnostic plots confirmed that all models’ assumptions had been met.

**Table 2:** Correlation Matrix of Total Sample, Including Typically Developing and Autistic Children

	1	2	3	4	5	6	7	8
1. Age	–	.07	.01	.41*	.09	.01	− .43*	.13
2. IQ		–	.01	.32*	.02	.11	− .01	− .12
3. AQ-Child			–	− .26	− .16	− .77*	.08	.66*
4. KEDS				–	.17	.35*	− .24	− .23
5. EmQue-CA					–	.46*	− .34*	− .30*
6. EQ						–	− .18	− .71*
7. AlexQ-C							–	.05
8. AlexQ-CP								–

*KEDS*: Kids Empathic Development Scale; *EMQue-CA*: Empathy Questionnaire for Children and Adolescents; *EQ*: Empathy Quotient; *AQ–Child:* Autism Quotient – Child; *IQ*: Intelligence Quotient; *AlexQ-CP*: Alexithymia Questionnaire for Children – Parent; *AlexQ-C*: Alexithymia Questionnaire for Children

*Significant at *p* < .05

**Table 3:** Correlation Matrix of Total Sample, Including typically developing children only

	1	2	3	4	5	6	7	8
1. Age	–	.09	− .10	.45*	.10	.06	− .43*	.08
2. IQ		–	.10	.29*	.00	.04	.05	− .05
3. AQ-Child			–	− .21	− .12	− .74*	.05	.59*
4. KEDS				–	.12	.29*	− .15	− .13
5. EmQue-CA					–	.44*	− .33*	− .26
6. EQ						–	− .14	− .66*
7. AlexQ-C							–	− .02
8. AlexQ-CP								–

KEDS: Kids Empathic Development Scale; EMQue-CA: Empathy Questionnaire for Children and Adolescents; EQ: Empathy Quotient; AQ – Child: Autism Quotient – Child; IQ: Intelligence Quotient; AlexQ-CP: Alexithymia Questionnaire for Children – Parent; AlexQ-C: Alexithymia Questionnaire for Children. *Significant at *p* < .05

### Relationship Between Empathy, Alexithymia and Autistic Traits

To identify the significant predictor(s) of empathy, a hierarchical multiple linear regression model with the empathy index as the outcome, the covariates age, gender and IQ and the experimental variables the alexithymia index and the AQ-Child as predictors was built. The first model, only including covariates, did not account for a significant amount of variance (*F*(3,55) = 2.55, *p* < 0.09), but the addition of the alexithymia index and the AQ-Child in the second step resulted in a model that performed above chance level in predicting empathic ability (*F*(5,52) = 9.62, *p* < 0.01). The R-squared value (0.48) indicated that this regression model accounted for 48% of the variability in the outcome measure (adjusted R^2^ = 0.43). Unlike age (*β* = 0.12, *t*(52) = 1.13, *p* = 0.27), gender (*β* = -0.110, *t*(52) = -0.50, *p* = 0.62) and IQ (*β* = 0.17, *t*(52) = 1.64, *p* = 0.11); alexithymia (*β* = -0.42, *t*(52) = -3.50, *p* < 0.01) as well as autistic traits *(β* = -0.33, *t*(52) = -2.62, *p* < 0.05) were significant predictors of empathy z-scores. After controlling for multiple comparisons using False Discovery Rate with *q* < 0.05 however, alexithymia scores remained the only significant predictor (Bonferroni corrections: *q* < 0.01, Holm corrections: *q* < 0.01), as autistic traits were no longer significant (Bonferroni: *q* = 0.07, Holm: *q* = 0.06). Cohen’s f^2^ was 0.93 for alexithymia, indicating a large effect size.

In addition to running a hierarchical multiple regression with the Empathy index as the outcome variable, separate models for the three different empathy measures (i.e., KEDS, EQ and EmQue-CA) were run to investigate alexithymia’s predictive ability on self-reported, parent-reported and behavioural-task measured empathic behaviour. Furthermore, the two measures of alexithymia, child- and parent-report were also analysed separately. As F-tests showed that gender did not significantly improve model fit for any of the models predicting empathy, gender was not included as a predictor for these models.

### Relationship Between Behavioural Empathy, Alexithymia and Autistic Traits

First, a model only including covariates was built to predict KEDS scores indicating that IQ and age explained 25% of the variance (*F*(2,55) = 9.17, p < 0.01; R^2^ = 0.25; adjusted R^2^ = 0.22). In a second step, AQ-Child was added as a predictors (*F*(3,53) = 8.18, p < 0.01; R^2^ = 0.32; adjusted R^2^ = 0.28; Cohen’s f^2^ = 0.47) with results indicating that AQ-Child was a significant predictor of KEDS scores (β = -0.26, t(53) = -2.28, p < 0.03). However, this effect was attenuated to non-significance when adding AlexQ-CP as a predictor in a third step. The final model (*F*(4,51) = 6.43, p < 0.01; R^2^ = 0.34; adjusted R^2^ = 0.28; Cohen’s f^2^ = 0.52) for the behavioural empathy measure (total KEDS score) indicated that neither AlexQ-CP (β = -0.14, t(51) = -0.86, p = 0.40) nor AQ-Child (β = -0.16, t(51) = -1.01, p = 0.32) significantly predicted behavioural empathy, but an ANOVA of the full model (Table 4) showed that while adding AQ-Child did not significantly improve the model, adding parent-reported alexithymia as a predictor significantly improved the model fit. This suggests that alexithymia may be a better predictor of empathy abilities than AQ-Child when IQ and age are taken into account. Specifically, lower alexithymia scores predicted more advanced empathy abilities. Child alexithymia questionnaire scores did not significantly improve model fit in a model that included age, IQ, AlexQ-C and AQ-Child (*F*(1,55) = 0.54, p = 0.47).

**Table 4:** Analysis of variance on KEDs score

	Df	Sum Sq	Mean Sq	F-value	2η	p-value
Age	1	9.62	9.62	13.05	.17	< .01*
IQ	1	5.10	5.10	6.92	.09	< .02*
AlexQ-CP	1	3.49	3.49	4.74	.06	< .04*
AQ-Child	1	0.76	0.76	1.03	.01	0.32
Residuals	51	37.60	0.74		.65	

IQ: Intelligence Quotient; AlexQ-CP: Alexithymia Questionnaire for Children – Parent; AQ-Child: Autism Quotient – Child. The ANOVA shows that, while adding the AlexQ-CP as a predictor significantly improves the model that includes age and IQ, inserting AQ does not significantly improve a model that already includes AlexQ-CP. *Significant at *p* < .05

### Relationship Between Self-Reported Empathy, Alexithymia and Autistic Traits

As a first step, a model only including covariates was built indicating that the model was not a good fit for the data (*F*(2,56) = 0.21, p = 0.82). Entering AQ-Child in a second step also did not result in a good fitting model (*F*(2,55) = 0.62, p = 0.61) with AQ-Child not being a significant predictor of self-reported empathy (β = -0.17, t(54) = -1.24, p = 0.22). In a model that further adjusted for alexithymia (*F*(4,52) = 1.62, p = 0.18; R^2^ = 0.11; adjusted R^2^ = 0.04; Cohen’s f^2^ = 0.12); AQ-Child was also not a significant predictor (β = 0.09, t(52) = 0.50, p = 0.62), whereas the AlexQ-CP significantly negatively predicted EmQue-CA scores (β = -0.38, t(52) = -2.12, p = 0.04). However, this association was no longer significant after correcting for multiple comparisons (Bonferroni: *q* = 0.19, Holm: *q* = 0.19). Overall, the model was not a good fit for the data (p = 0.18). The ANOVA (Table 5) of the full model, which included the outcome EmQue-CA and the predictors age, IQ, AlexQ-CP and AQ-Child, showed again that adding AlexQ-CP as a predictor significantly improved model fit while adding AQ-Child did not improve a model that already included the AlexQ-CP.

**Table 5:** Analysis of variance on EmQue-CA score, with parent-reported alexithymia as predictor

	*Df*	*Sum Sq*	*Mean Sq*	*F*-value	2*η*	*p*-value
Age	1	0.35	0.35	0.36	.01	.55
IQ	1	0.06	0.06	0.06	.01	.80
AlexQ-CP	1	5.66	5.66	5.81	.10	< .02*
AQ-Child	1	0.24	0.24	0.25	.01	.62
Residuals	52	50.72	0.98	.89		

*Note*. IQ: Intelligence Quotient; AlexQ-CP: Alexithymia Questionnaire for Children – Parent; AQ-Child: Autism Quotient – Child. The ANOVA shows that, while adding AlexQ-CP as a predictor significantly improves a model that includes age and IQ, adding AQ does not significantly improve a model that already includes the AlexQ-CP. *Significant at *p* < .05

A model using the self-report of alexithymia as a predictor instead of the parent-reported alexithymia replicated the same pattern (Table 6), with the overall model not fitting well (*F*(4,53) = 2.21, p = 0.18; R^2^ = 0.14; adjusted R^2^ = 0.08; Cohen’s f^2^ = 0.16;) and self-reported alexithymia emerging as the only significant predictor (β = -0.36, t(53) = -2.60, p = 0.01, Bonferroni: *q* = 0.06, Holm: *q* = 0.06).

**Table 6:** Analysis of variance on EmQue-CA score, with self-reported alexithymia as predictor

	*Df*	*Sum Sq*	*Mean Sq*	*F*-value	2*η*	*p*-value
Age	1	0.31	0.31	0.33	.01	.57
IQ	1	0.03	0.03	0.04	.01	.85
AlexQ-C	1	6.82	6.82	7.32	.12	< .01*
AQ-Child	1	1.07	1.07	1.15	.02	.29
Residuals	53	49.40	0.93		.86	

IQ: Intelligence Quotient; AlexQ-CP: Alexithymia Questionnaire for Children – Parent; AQ-Child: Autism Quotient – Child. The ANOVA shows that, while adding AlexQ-CP as a predictor significantly improves a model that includes age and IQ, adding AQ-Child does not significantly improve a model that already includes the AlexQ-CP. *Significant at *p* < .05

### Relationship between Parent-Reported Empathy, Alexithymia and Autistic Traits

Again, the first model only included covariates (*F*(2,55) = 0.33, p = 0.72) with AQ-child (*F*(3,53) = 27.52, p =  < 0.01) and the AlexQ-CP being entered in subsequent steps. A model (*F*(4,51) = 27.71, p < 0.01; R^2^: = 0.69; adjusted R^2^: = 0.66; Cohen’s f^2^: = 2.23) that included EQ as the outcome and age, IQ, AlexQ-CP and AQ-Child as predictors showed that the AlexQ-CP (β = − 0.36, t(51) = -3.36, p < 0.01) as well as AQ-Child (β = − 0.54, t(51) =  − 5.14, p < 0.01) significantly negatively predicted the EQ score. These results were still significant after adjusting for multiple comparisons (Bonferroni corrections: *q* < 0.01, Holm corrections: *q* < 0.01). In a model that included the self-report of alexithymia instead of the parent-report of alexithymia as a predictor (*F*(4,52) = 21.80, p < 0.01; R^2^: = 0.63; adjusted R^2^: = 0.6; Cohen’s f^2^: = 1.71), only AQ-Child was a significant predictor (β = -0.77, t(52) =  − 8.95, p < 0.01, Bonferroni corrections: *q* < 0.01, Holm corrections: *q* < 0.01). An ANOVA, however, again showed that adding the AlexQ-C as a predictor significantly improved the model (F(1,55) = 80.01, p < 0.03). Hence, for parent measures of empathy, the AQ-Child was still a strong predictor of empathy abilities even after adjusting for alexithymia.

## Discussion

The present study is the first to examine the relationships between empathy, autistic traits and alexithymia in preadolescent children. As hypothesised, alexithymia significantly predicted empathy challenges in children. In addition, results suggested that alexithymia is a more powerful predictor of empathy abilities than autistic traits, consistent with the theory that socio-emotional difficulties classically associated with ASC may be explained by co-occurring alexithymia. The findings found preliminary evidence for the alexithymia-hypothesis (Bird & Cook, 2013) in children; previously only supported in the majority of adult populations.

The results of the current study are also consistent with the shared network model of empathy theory (Singer et al., 2004), which proposes the emotions of both the self and of others are processed by the same neural networks (Bird et al., 2010; Fan et al., 2011). As such, it appears that difficulties in identifying and describing one’s own emotions may lead to difficulties in representing others’ emotions, supporting the work of Bird and Viding (2014). Indeed, Fletcher-Watson and Bird (2019) contested the notion that autistic individuals experience difficulties in resonating with, understanding and having an affinity towards others’ emotional reactions. The authors argued that this challenge that some autistic individuals experience is better explained by their potentially underlying alexithymic traits. Thus, the findings from the current study are in line with Fletcher-Watson and Bird’s (2019) speculation.

We also found that parent- and self-reported alexithymia scores were not significantly correlated and that it was the latter that was the main predictor of decreased empathy until multiple comparisons were corrected for. The lack of association between alexithymia reports from different informants was similar to the findings of Griffin and colleagues (2015) and is consistent with findings in child mental health research more broadly, which show that child- and parent-reports of psychological phenomena often fail to correlate significantly (De Los Reyes & Kazdin, 2005; De Los Reyes et al., 2015). While there has been little research on informant discrepancies in relation to alexithymia specifically, much work in other areas of child mental health has suggested that this reflects the fact that different informants capture different but reliable variance in child symptoms (e.g. De Los Reyes, 2011; Brown et al., 2020). However, further work evaluating informant discrepancies in alexithymia would be valuable for understanding the direction and predictors of disagreements between informants. In both research and clinical contexts, the inclusion of data from multiple informants is considered good practice in order to obtain a fuller picture of the child’s functioning across different contexts. Our results suggest that children’s self-reports could offer valuable insights into their awareness of their emotional strengths and challenges. For example, the inclusion of self-reports may allow clinicians to gain a better understanding of the child’s own interpretation of their emotions and, hence, allow them to target the child’s specific difficulties. This finding further supports the inclusion of multiple informants when assessing a child’s emotional processing abilities.

The links between alexithymia and empathy further point to the potential value of interventions that increase emotional awareness for individuals both with and without autism to help improve empathy. There is, for example, growing evidence of the benefits of mindfulness-based interventions for enhancing empathy (Birnie et al., 2010; Norman et al., 2019). Even though the literature in child populations is limited, mindfulness-based interventions have been successfully used in autistic children (Hwang et al., 2015) and adolescents (de Bruin et al., 2015) to improve prosocial behaviours. It has been suggested that practicing mindfulness can aid individuals to become more perceptive of their own emotions, in turn facilitating a heightened awareness of others’ emotional states (Birnie et al., 2010), implying that these interventions may work via ameliorating alexithymic traits. Taken together, the results of the current study suggest autistic children, who screen positive for co-occurring alexithymic traits may benefit from interventions such as mindfulness-based interventions.

### Limitations and Future Work

There are some limitations to consider. First, as our sample size was a relatively small convenience sample, the findings should be replicated in studies with sampling designs that will permit generalisations to well-defined relevant populations. Second, the current study only utilised one experimental task when assessing child empathy. While the KEDS offered insight into the child’s cognitive, affective, and behavioural empathy, it may be beneficial for future research to administer additional tasks that assess other empathic processes (e.g. theory of mind, experiencing empathic concern for others and/or identifying emotion from vocal and facial stimuli). Third, variance in the significant predictive abilities of alexithymia was observed across the current study’s regression analyses. As described previously, cross-informant discrepancies are typically observed in measures of child behaviour and psychopathology (De Los Reyes & Kazdin, 2005). Thus, relying on a singular measure may provide insufficient information for it to emerge as a significant predictive variable, as seen in some of the current study’s regression analyses. Thus, the findings from the current study bolster the notion for collecting data across multiple-informants when investigating child empathy. Indeed, when combining the child- and parent-reported alexithymia scores, the alexithymia index emerged as a significant predictor of child empathic difficulties. Nonetheless, the results from the current study should be considered preliminary and replication is required, particularly in larger sample sizes. Fourth, future studies could leverage longitudinal data to examine the developmental relations between alexithymia, empathy, and autistic traits to establish how symptoms in these three domains may influence one another over time and to establish critical periods where interventions may be most important to target. Finally, it would also be desirable to adapt the tasks to make them applicable to autistic children with higher-support needs to help address the under-representation of these children in autism research (Stedman et al., 2018).

## Conclusion

Our findings suggest that alexithymia and not autistic traits primarily predict empathic difficulties in preadolescent children. These results provide support for the ‘alexithymia hypothesis’ (Bird & Cook, 2013) in younger populations, which was previously only supported by findings from older populations (e.g. Cook et al., 2013). From a therapeutic perspective, results suggest that autistic children who screen positive for elevated alexithymic traits may benefit from additional support during intervention strategies. For example, incorporating mindfulness-based interventions during the child’s treatment may help ameliorate their co-occurring alexithymic traits, in turn potentially improving response to empathy-specific interventions.
